# Percepción estética de la sonrisa según la exposición gingival en estudiantes universitarios de Lima, Perú, 2020

**DOI:** 10.21142/2523-2754-0904-2021-081

**Published:** 2021-12-09

**Authors:** Mery Angela Abadíe Miranda, Jocelyn Graciela Lugo-Varillas, Úrsula María Dolores Albites Achata

**Affiliations:** 1 Carrera de Estomatología, Facultad de Ciencias de la Salud, Universidad Científica del Sur. Lima, Perú. meryangela7@gmail.com, jocelyn.lugo.varillas@gmail.com, ursulaalbites@gmail.com Universidad Científica del Sur Carrera de Estomatología Facultad de Ciencias de la Salud Universidad Científica del Sur Lima Peru meryangela7@gmail.com jocelyn.lugo.varillas@gmail.com ursulaalbites@gmail.com

**Keywords:** percepción, sonrisa, encía, estética, perception, smile, gingiva, esthetics

## Abstract

**Objetivo:**

Determinar la percepción estética de la sonrisa según exposición gingival en estudiantes universitarios de odontología y otras carreras (Lima, Perú 2020); evaluar de manera descriptiva las variables principales, como estudiante, edad y sexo; y comparar la percepción estética la sonrisa según exposición gingival entre estudiantes universitarios de pregrado, diferentes edades y sexos.

**Metodología:**

Se fotografió la sonrisa de una mujer siguiendo los parámetros estéticos, se modificó digitalmente la exposición gingival, para obtener seis imágenes digitales. Fueron calificadas por 512 estudiantes de odontología y otras carreras, mediante una encuesta virtual utilizando la escala analógica visual (valores 0 al 10).

**Resultados:**

Estudiantes universitarios percibieron la sonrisa con 0,5 mm de exposición gingival como más atractiva y la sonrisa con 2,5 mm de exposición gingival como poco atractiva. Según la edad, los estudiantes de 18 a 23 años percibieron más atractiva la sonrisa con 0,5 mm exposición gingival y menos agradable la sonrisa 2,5 mm exposición gingival, los estudiantes de 24 a 29 años percibieron como sonrisa más atractiva con 1mm exposición gingival y poco atractiva la sonrisa 2mm exposición gingival. Según el sexo, a ambos les pareció más atractiva la sonrisa 0,5 mm exposición gingival y poco atractiva la sonrisa con 2,5 mm de exposición gingival.

**Conclusiones:**

Estudiantes universitarios de odontología y otras carreras tuvieron la misma percepción de la sonrisa con relación a la exposición gingival, percibieron atractiva la sonrisa con 0,5 mm de exposición gingival y poco atractiva la que tiene 2,5 mm. Estudiantes más jóvenes consideraron más agradable la sonrisa con exposición gingival de 0,5 mm. Estudiantes de ambos sexos tuvieron una misma percepción estética de la sonrisa.

## Abstract (en)

**Objective:**

To determine the esthetic perception of the smile according to gingival exposure in undergraduate university students of dentistry and other degrees of different ages and sex in Lima, Peru 2020, and descriptively evaluate the main variables such as age and sex.

**Methodology:**

A woman's smile was photographed following esthetic parameters, digitally modifying gingival exposure and obtaining six digital images. The images were rated by 512 students of dentistry and other degrees by means of a virtual survey using the Visual Analog Scale (values 0 to 10).

**Results:**

The students perceived the 0.5 mm gingival exposure smile as more attractive and the 2.5 mm gingival exposure smile as unattractive. According to age, students aged 18 to 23 years perceived the 0.5 mm gingival exposure smile as more attractive and the 2.5 mm gingival exposure smile as less attractive, while students aged 24 to 29 years perceived the 1 mm gingival exposure smile as more attractive and the 2 mm gingival exposure smile as unattractive. According to gender, both found the 0.5 mm gingival exposure smile more attractive and the 2.5mm gingival exposure smile unattractive.

**Conclusions:**

University students of dentistry and other degrees had the same perception of the smile in relation to gingival exposure, with a smile of 0.5 mm gingival exposure being considered attractive and the 2.5 mm smile unattractive. Younger students considered the smile with 0.5 mm gingival exposure more pleasant. Students of both sexes had the same esthetic perception of the smile.

## INTRODUCCIÓN

En la actualidad, la estética se ha convertido en un factor muy importante en la vida de las personas. La sonrisa es una de ellas, debido que juega un rol esencial en la apariencia y la autoestima. De tal manera, si no se encuentra dentro de los parámetros socialmente aceptados como “estéticos”, puede provocar efectos psicosociales negativos, entre ellos la falta de habilidades para socializar ^1^^,^^2^.

La boca es el centro de comunicación en la cara, de modo que genera interés para otras personas; es así como el grado de satisfacción de los pacientes se hallará íntimamente relacionado con parámetros de la estética como forma de labios, línea de la sonrisa, posición de los dientes y visibilidad de las encías, lo que le da proporcionalidad y armonía al rostro ^3^^,^^4^. La sonrisa es empleada para trasmitir diferentes aspectos de la vida de las personas, como emociones positivas o intenciones sociales ^5^^,^^6^.

La percepción estética varía en cada persona y está influenciada por las experiencias personales, ambientales y sociales ^7^^-^^9^. Los pacientes llegan a la consulta con el propósito de mejorar la estética de su sonrisa para verse y sentirse mejor, por lo cual es imprescindible brindarles una sonrisa armoniosa, agradable y equilibrada a la vista de los pacientes, sin afectar su percepción ^5^^,^^7^^,^^10^.

Durante la última década, la estética facial y dental ha cobrado importancia debido a que los pacientes llegan con un concepto de estética preconcebido y, a su vez, los clínicos buscan realizar un diagnóstico y planificación del tratamiento orientado a satisfacer las expectativas del paciente ^10^^,^^11^. Entre los diferentes parámetros estéticos de la sonrisa, se pueden distinguir tres aspectos esenciales: cantidad de exposición gingival, arco de sonrisa y tamaño de corredor bucal ^7^^,^^10^.

Por otro lado, es importante conocer la etiología de la sonrisa gingival, como el crecimiento vertical excesivo de la maxila, la erupción pasiva retrasada, la hiperelevación muscular del labio superior, la longitud de la corona clínica corta, la extrusión maxilar dentoalveolar, la hipermovilidad labial y un labio corto, reflejado en una incompetencia labial ^12^^-^^14^. Una mayor cantidad de exposición gingival puede desfavorecer la conformidad de la apariencia de la sonrisa de acuerdo con los estándares de simetría facial ^15^^-^^17^. Se estima una sonrisa gingival cuando los individuos exponen tejido gingival, y al medir es mayor a 3 mm, lo cual puede ser percibido por una persona sin conocimientos sobre parámetros estéticos de la sonrisa ^15^^,^^18^^,^^19^.

Existen estudios que señalan que los estudiantes de odontología tienen mayor análisis crítico estético sobre la percepción estética de la sonrisa que las poblaciones que no lo tienen ^6^^,^^20^. Además, otros estudios realizaron comparaciones en personas sin conocimientos sobre parámetros estéticos de la sonrisa basándose en las diferentes edades, y hallaron que la percepción de la sonrisa tiene un mayor impacto en los jóvenes que en los adultos ^4^^,^^20^. Asimismo, estudios sobre la influencia en el sexo y el impacto en las percepciones orofaciales señalan que las mujeres presentan una mejor visión de la encía en comparación con los varones ^21^^,^^22^.

El presente estudio busca generar un conocimiento sobre un juicio crítico de la percepción de la sonrisa según exposición gingival en estudiantes odontólogos y no odontólogos de diferentes edades y sexos. Además, estandarizar criterios en estudiantes de odontología, sobre todo en la etapa de experiencia clínica, con relación a la percepción de una sonrisa armoniosa considerando la exposición gingival, con lo que se obtuvo un trabajo multidisciplinario bajo un mismo criterio durante su etapa de formación. 

Por tal motivo, el propósito del estudio fue evaluar la percepción estética de la sonrisa según la exposición gingival de acuerdo con el tipo de estudiante, el sexo y la edad en universitarios de Lima, en el año 2020. 

## MATERIALES Y MÉTODOS

El estudio fue observacional descriptivo, transversal y prospectivo. El tamaño muestral se obtuvo utilizando la fórmula de comparación de medias con un poder estadístico del 90% y un nivel de confianza del 95%, lo que dio como resultado 196 muestras por grupo, y se decidió aumentar 60 muestras por grupo para llegar a toda la población. En total, la muestra fue de 512 estudiantes universitarios.

La muestra consistió en alumnos de pregrado de la Universidad Científica del Sur (Lima-Perú), de la carrera de Estomatología, en el año 2020, y alumnos de pregrado sin discernimientos sobre parámetros estéticos de la sonrisa de una carrera diferente a la de Estomatología y de otras universidades de Lima (Perú). Se incluyeron estudiantes universitarios de sexo femenino y masculino con edades entre 18 y 29 años que firmaron el consentimiento informado, aceptando su participación en el estudio.

Se inició solicitando la autorización al director de carrera de Estomatología de la Universidad Científica del Sur, donde se realizó el estudio. Después de obtener la aprobación del Comité Institucional de Ética en Investigación de la Universidad Científica del Sur y el código de aprobación 659-2019-PRE8, se elaboró un consentimiento informado estableciendo en qué consiste el estudio y la aceptación de los encuestados. El encuestado colocó sus datos personales, edad, sexo y ciclo en el que se encuentra. Asimismo, las instrucciones para el llenado de la encuesta y la evaluación de cada fotografía mediante la Escala Visual Análoga (VAS) tuvieron valores de 0-10, según los cuales 0-5 representó poco agradable y 6-10, muy agradable ^23^.

**Figura 1:**
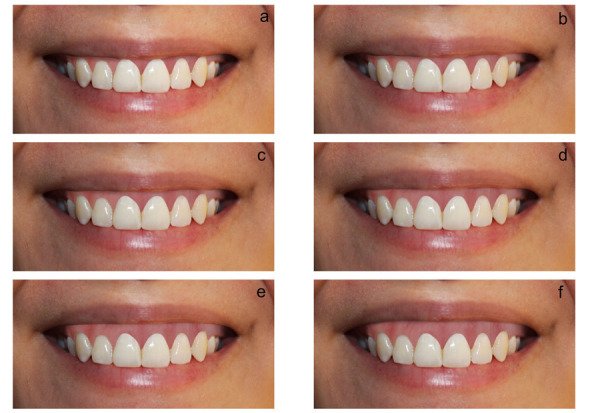
Sonrisa con diferentes niveles de exposición gingival: **a)** sonrisa con exposición gingival de 0 mm; **b)** sonrisa con exposición gingival de 0,5 mm; **c)** sonrisa con exposición gingival de 1 mm; **d)** sonrisa con exposición gingival de 1,5 mm; **e)** sonrisa con exposición gingival de 2 mm; **f)** sonrisa con exposición gingival de 2,5 mm.

Un fotógrafo profesional tomó la imagen de la sonrisa de una mujer con patrones estéticos y armoniosos a la visión, a quien se le entregó una carta de consentimiento informado. La modelo estuvo de pie, con la cabeza en posición natural y paralela al piso, enfocando solo la sonrisa. La imagen fue alterada con el programa Adobe Photoshop CS5, con la ayuda de un experto, para crear condiciones periodontales de la sonrisa exponiendo encía con altura de 0,5 mm a 2,5 mm, añadidas a la fotografía original. Se obtuvo seis imágenes, como se muestra en la Figura 1.

Para la evaluación de las fotografías, se usó una encuesta virtual mediante el programa Google Forms, enviada mediante un enlace durante las clases a los alumnos de la carrera de Estomatología y a los alumnos de otras universidades. 

Mediante el programa estadístico SPSS versión 23 (SPSS Inc. Chicago, Ill, EE. UU.), se realizó el análisis descriptivo para conocer frecuencias y proporciones de las variables evaluadas tales como estudiantes, edad y sexo. Posteriormente, se utilizó la prueba Kolmogórov-Smirnov para evaluar la distribución de datos, luego se utilizó la prueba U de Mann-Whitney para evaluar las diferencias significativas en ambos grupos. Todo se trabajó con un nivel de significancia de p < 0,05.

## RESULTADOS

Luego de realizar las encuestas se obtuvo una cantidad de 256 (50%) estudiantes de odontología y 256 (50%) de otras carreras, lo que da un total de 512 estudiantes universitarios. Los estudiantes fueron divididos según edad y sexo. Según edad, se dividió en dos grupos: 440 estudiantes entre 18 y 23 años (85,90%) y 72 estudiantes entre 24 y 29 años (14,10%); según sexo: 349 del femenino (68,20%) y 163 del masculino (31,80%) (Tabla 1).

**Tabla 1:** Evaluación descriptiva de las variables principales del estudio con relación a la percepción estética de la sonrisa según la exposición gingival: estudiantes, edad y sexo

Variables		N	
		f	%
Estudiantes	Estudiantes de odontología	256	50%
	Estudiantes de otras carreras	256	50%
Edad	18-23 años	440	85,90%
	24-29 años	72	14,10%
Sexo	Femenino	349	68,20%
	Masculino	163	31,80%

La Tabla 2 corresponde a la comparación de la percepción estética de la sonrisa según la exposición gingival en estudiantes universitarios de pregrado; los estudiantes de odontología y otras carreras percibieron que la pregunta 4 (exposición gingival 0,5 mm) más atractiva en cuanto a la media fue 5,41 y 6,18, respectivamente; mientras que la pregunta 6 (exposición gingival 2,5 mm) presenta menor percepción atractiva en cuanto al promedio, obtuvieron una media de 2,35 y 4,23, respectivamente. Se observaron promedios más altos en los estudiantes de odontología que en los de otras carreras. En cuanto a la diferencia de percepción, se observó que, a mayor exposición gingival, hay mayor diferencia de percepción estética de la sonrisa gingival; en cuanto al promedio, estos resultados fueron estadísticamente significativos (p < 0,05).

**Tabla 2:** Comparación de la percepción estética de la sonrisa según la exposición gingival en estudiantes universitarios de odontología y otras carreras

Exposición gingival	Estudiante de odontología		Estudiantes de otras carreras		p	Diferencia de percepción*
	Media	DE	Media	DE		
Pregunta 1 (0 mm)	4,81	2,61	5,7	2,2	<0,001	51,56
Pregunta 2 (1 mm)	4,31	2,34	5,54	2,09	<0,001	79,38
Pregunta 3 (2 mm)	3,05	2,24	4,7	2,31	<0,001	104,5
Pregunta 4 (0,5 mm)	5,41	2,35	6,18	1,96	<0,001	47,96
Pregunta 5 (1,5 mm)	3,69	2,23	5,27	2,21	<0,001	101,72
Pregunta 6 (2,5 mm)	2,35	2,28	4,23	2,46	<0,001	111,76

*Prueba de U-Mann Whitney (p<0.05)

En la Tabla 3 se observa la comparación de la percepción estética de la sonrisa según la exposición gingival en estudiantes universitarios de pregrado, según la edad, en la que no se encontraron diferencias estadísticamente significativas (p > 0,05). Se observaron promedios más altos en los estudiantes más jóvenes en ambos grupos. Con respecto a la diferencia de percepción entre los 2 rangos de edades (18-23 y 24-29), los estudiantes de otras carreras tuvieron una mayor diferencia de percepción que los estudiantes de odontología, en cuanto al promedio.

**Tabla 3:** Comparación de la percepción estética de la sonrisa según la exposición gingival en estudiantes universitarios de pregrado, según la edad

Estudiantes	Estudiantes de odontología						Estudiantes de otras carreras					
Edad	18-23 años		24-29 años		p	Diferencia de percepción*	18-23 años		24-29 años		p	Diferencia de percepción*
Exposición gingival	Media	DE	Media	DE			Media	DE	Media	DE		
Pregunta 1 (0 mm)	4,87	2,6	4,57	2,66	0,55	7,05	5,76	2,2	5,2	2,12	0,21	19,06
Pregunta 2 (1 mm)	4,37	2,34	4,02	2,37	0,36	10,64	5,52	2,12	5,64	1,82	0,84	-2,95
Pregunta 3 (2 mm)	3,05	2,24	3,06	2,26	0,75	3,76	4,71	2,31	4,6	2,36	0,75	4,77
Pregunta 4 (0,5 mm)	5,43	2,34	5,3	2,41	0,76	3,63	6,26	1,95	5,48	2	0,05	29,72
Pregunta 5 (1,5 mm)	3,71	2,25	3,6	2,8	0,72	4,17	5,3	2,22	5	2,14	0,41	12,5
Pregunta 6 (2,5 mm)	2,4	2,3	2,11	2,16	0,43	9,22	4,18	2,49	4,64	2,23	0,31	-15,43

*Prueba de U-Mann Whitney (p<0,05)

La Tabla 4 corresponde a la comparación de la percepción estética de la sonrisa según la exposición gingival en estudiantes universitarios de pregrado, según el sexo, no se encontraron diferencias estadísticamente significativas (p > 0,05). Los estudiantes de odontología de ambos sexos obtuvieron la media más alta de 5,41 en la pregunta 4 (exposición gingival de 0,5 mm), mientras que los estudiantes de otras carreras de sexo femenino y masculino obtuvieron una media más alta de 6,25 y 6,02, respectivamente. Con relación a la diferencia de percepción, no se encontraron diferencias marcadas entre ambos grupos.

**Tabla 4:** Comparación de la percepción estética de la sonrisa según la exposición gingival en estudiantes universitarios de pregrado, según el sexo.

Estudiantes	Estudiantes de odontología						Estudiantes de otras carreras					
Sexo	Femenino		Masculino		p	Diferencia de percepción*	Femenino		Masculino		p	Diferencia de percepción*
Exposición gingival	Media	DE	Media	DE			Media	DE	Media	DE		
Pregunta 1 (0 mm)	4,81	2,66	4,82	2,51	0,80	2,52	5,65	2,26	5,8	2,09	0,76	-2,84
Pregunta 2 (1 mm)	4,26	2,41	4,43	2,17	0,60	-5,3	5,44	2,08	5,72	2,11	0,50	-6,48
Pregunta 3 (2 mm)	2,94	2,26	3,32	2,19	0,22	-12,29	4,74	2,3	4,61	2,34	0,58	5,26
Pregunta 4 (0,5 mm)	5,41	2,41	5,41	2,21	0,77	2,95	6,26	1,97	6,02	1,95	0,29	10,01
Pregunta 5 (1,5 mm)	3,59	2,31	3,93	2,02	0,27	-10,94	5,23	2,17	5,34	2,3	0,67	-4,02
Pregunta 6 (2,5 mm)	2,29	2,31	2,49	2,21	0,37	-9,47	4,26	2,55	4,16	2,31	0,77	2,78

*Prueba de U-Mann Whitney (p<0,05)

## DISCUSIÓN

El propósito de estudiar la percepción de estudiantes universitarios de odontología y de otras carreras de diferentes edades y sexo radica en comprender el juicio crítico de la percepción de la sonrisa según la exposición gingival, y a la vez tener un mismo criterio en beneficio del paciente al realizar determinados procedimientos que requieran conocimientos de los parámetros estéticos en la sonrisa.

Al asociar la percepción estética de la sonrisa según la exposición gingival en estudiantes de odontología y otras carreras, se encontró que hubo una diferencia significativa; en contraste, el estudio de Pithon *et al*. ^24^ realizó una encuesta entre personas sin conocimiento odontológico, odontólogos y estudiantes de odontología, y encontró que las diferencias no fueron estadísticamente significativas a pesar de que los grupos coincidieron con la fotografía que les pareció más agradable y la menos agradable, probablemente se deba que la población del presente estudio fue mayor.

Se encontró que la fotografía con exposición gingival de 0,5 mm fue percibida como la más agradable y la fotografía con exposición gingival de 2,5 mm como la menos agradable, lo cual demostró que los estudiantes de otras carreras tienden a tener una menor percepción estética. Otros estudios ^25^^,^^26^ evaluaron la percepción de la sonrisa en odontólogos y no odontólogos, y mostraron que las personas con conocimiento odontológico encontraron más agradable una sonrisa con exposición gingival de 0 mm. Cabe mencionar que estos estudios se realizaron en poblaciones diferentes a la nuestra (China y Brasil), y la diferencia de resultados entre ambos estudios podría deberse a que existen distintas maneras de ver la estética de la sonrisa en los grupos étnicos. 

Según los resultados obtenidos en la percepción estética de la sonrisa según la edad, no se demostró una diferencia estadísticamente significativa, el mismo resultado lo obtuvieron otros estudios ^18^^,^^27^. A su vez, en el estudio se encontró que los estudiantes universitarios más jóvenes obtuvieron promedios más bajos, en los que percibieron como más agradable la sonrisa con exposición gingival de 0,5 mm y la sonrisa con exposición gingival de 2,5 mm como la menos agradable. En el grupo de adultos jóvenes, los estudiantes de odontología percibieron como más agradable la sonrisa con exposición gingival de 0,5 mm y menos agradable la sonrisa con exposición gingival de 2,5 mm; a diferencia de los estudiantes de otras carreras, quienes percibieron que la sonrisa con exposición gingival de 1 mm era más agradable y como menos agradable la sonrisa con exposición gingival de 2 mm; estos resultados coinciden con el estudio realizado por Sriphdungporn *et al*. ^18^, probablemente se deba a que los más jóvenes tienen un juicio crítico mayor en cuanto a la percepción estética de la sonrisa a comparación de los de adultos jóvenes.

Entre los resultados obtenidos en este estudio se encontró que los estudiantes universitarios más jóvenes percibieron más agradable la fotografía de la sonrisa con exposición gingival de 0,5 mm, a diferencia de los resultados obtenidos en un estudio realizado por España *et al*. ^27^, el cual señala que los estudiantes de primer, segundo y último año percibieron como más agradable la fotografía que mostró una sonrisa con exposición gingival de 0 mm; lo cual indica que los estudiantes de odontología de España muestran una ligera preferencia por sonrisas con menor presencia de exposición gingival, en comparación con los estudiantes de odontología de la Universidad Científica del Sur de Lima, Perú.

Los resultados hallados en la percepción estética de la sonrisa según el sexo mostraron que no hubo diferencias estadísticamente significativas en ambos sexos, mismo resultado obtenido en el estudio realizado por Abu Alhaija *et al*. ^28^, quienes evaluaron la percepción de la sonrisa en ortodoncistas, practicantes generales y personas laicas de Jordania. Al mismo tiempo, en los resultados hallados en el estudio, se obtuvo que los estudiantes universitarios de ambos sexos encontraron que la fotografía con exposición gingival de 0,5 mm fue la más agradable, mientras que la fotografía con exposición gingival de 2,5 mm fue percibida como poco agradable, contrario a los resultados encontrados en un estudio anterior realizado por Geron *et al*. ^29^, quienes observaron que las participantes de sexo femenino tuvieron un promedio más alto que los participantes de sexo masculino. Posiblemente sea porque las estudiantes universitarias de sexo femenino fueron menos tolerantes a la exposición gingival y la población femenina encuestada fue mayor a la de sexo masculino. 

Las limitaciones del presente estudio fueron el uso de una sonrisa femenina como único modelo para la encuesta; además, la heterogeneidad de los participantes por género, edad y grado de instrucción para tener valores más proporcionales. Por otro lado, se recomienda realizar estudios que evalúen otras variables que podrían influir en la percepción como factor socioeconómico, factor cultural, entre otros. Asimismo, se recomienda ampliar el estudio a otras poblaciones que tengan conocimiento de estética tales como artes escénicas, fotografía, artes plásticas, comunicaciones, ilustración, humanidades, maquilladoras, entre otros, para obtener diferentes perspectivas sobre la estética de la sonrisa en carreras artísticas.

## CONCLUSIONES

En el estudio se encontraron diferencias estadísticamente significativas en cuanto a la percepción estética de la sonrisa según la exposición gingival en los estudiantes de odontología y de otras carreras. Los estudiantes de odontología y otras carreras tuvieron la misma percepción estética de la sonrisa con relación a la exposición gingival. Los estudiantes universitarios más jóvenes de ambos grupos tuvieron una preferencia por una sonrisa con exposición gingival de 0,5 mm, mientras que los estudiantes universitarios de ambos sexos tuvieron una misma percepción estética de la sonrisa.

## Fuente de financiamiento:

Autofinanciado
